# Artificial intelligence technologies for enhancing neurofunctionalities: a comprehensive review with applications in Alzheimer’s disease research

**DOI:** 10.3389/fnagi.2025.1609063

**Published:** 2025-08-15

**Authors:** Zhirong Gu, Bin Ge, Yuanyuan Wang, Yiping Gong, Mei Qi

**Affiliations:** ^1^Department of Pharmacy, Gansu Provincial People’s Hospital, Lanzhou, Gansu, China; ^2^School of Pharmacy, Gansu University of Traditional Chinese Medicine, Lanzhou, Gansu, China

**Keywords:** Alzheimer’s disease, artificial intelligence, machine learning, cognitive training, healthcare innovation

## Abstract

Alzheimer’s disease (AD) is a progressive neurodegenerative condition that impairs memory and cognition, presenting a growing global healthcare burden. Despite major research efforts, no cure exists, and treatments remain focused on symptom relief. This narrative review highlights recent advancements in artificial intelligence (AI), particularly machine learning (ML) and deep learning (DL), which enhance early diagnosis, predict disease progression, and support personalized treatment strategies. AI applications are reshaping healthcare by enabling early detection, predicting disease progression, and developing personalized treatment plans. In particular, AI’s ability to analyze complex datasets, including genetic and imaging data, has shown promise in identifying early biomarkers of AD. Additionally, AI-driven cognitive training and rehabilitation programs are emerging as effective tools to improve cognitive function and slow down the progression of cognitive impairment. The paper also discusses the potential of AI in drug discovery and clinical trial optimization, offering new avenues for the development of AD treatments. The paper emphasizes the need for ongoing interdisciplinary collaboration and regulatory oversight to harness AI’s full potential in transforming AD care and improving patient outcomes.

## Graphical Abstract

Advancements in Artificial intelligence technologies for enhancing neurofunctionalities: applications in Alzheimer’s disease research. 
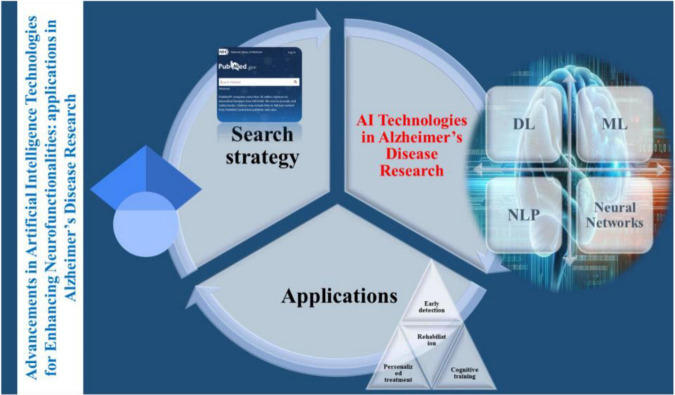

## 1 Introduction

Alzheimer’s disease is one of the leading neurodegenerative disorders characterized by progressive cognitive decline and memory loss. By contributing 60%–80% to global 55 million dementia cases, AD is currently the most common cause of dementia among older adult (Subasi et al., 2022). AD is marked by neuronal death and brain atrophy that is caused by the accumulation of amyloid-beta plaques and neurofibrillary tangles in the brain. Beginning with mild cognitive impairment, the disease progresses through several stages, finally advancing to severe dementia (Kiss et al., 2023). Despite major investments, such as the NIH allocating over $3.8 billion annually to AD research, the mechanisms of Alzheimer’s disease remain incompletely understood. No cure exists, and current therapies aim only to manage symptoms and slow disease progression (Bucholc et al., 2023).

Several challenges are faced in the timely diagnosis and treatment of AD, majorly due to the lack of specific biomarkers and subtle onset of symptoms. Current diagnostic methods that include cognitive assessments and imaging techniques can identify the disease at a later stage when significant brain damage has already occurred. Similarly, currently available therapeutics such as cholinesterase inhibitors and NMDA receptor antagonists are limited and primarily focus on symptomatic relief rather than disease modification (El-Sappagh et al., 2021). Due to limited understanding of AD pathophysiology and high clinical trial failure rates of clinical trials, the available medications do offer modest benefits but are unable to halt disease progression. This comprehension of complex molecular alterations leading to AD requires the integration of modern technology and complex machinery with the aim of understanding and developing novel therapeutics capable of mitigating the burden that AD is posing globally.

In recent years, AI has made significant growth majorly driven by advanced computational power, improved algorithm development, and the availability of multi-aspect large datasets. Nowadays, AI employs a broad range of technologies and methodologies that have been designed to enable machines to perform tasks that typically require advanced human intelligence (Bazarbekov et al., 2024). These tasks include complex learning, improved reasoning, advanced problem-solving, enhanced perception, and understanding and overcoming language barriers. Based on the mode of applications, AI technologies can be categorized into various subfields, including machine learning, deep learning, natural language processing, and computer vision. At its base, AI is defined as the capability of a machine to imitate human intelligence and behavior through the development of algorithms and models that allow machines to process information, learn from data, make decisions, and perform supervised actions autonomously. Due to these capabilities, the scope of AI spans numerous applications, from simple rule-based systems to sophisticated neural networks that can analyze complex data patterns. For instance, a subset of AI called Machine learning (ML) focuses on the development of algorithms that enable computers not only to learn from the data but also to make accurate predictions based on a pattern that the algorithm perceives (Kale et al., 2024). Similarly, another specialization of AI is Deep learning, which is an advanced form of ML utilizing neural networks with many layers to analyze complex datasets to model intricate data relationships (Swargiary, 2024).

## 2 Study eligibility and selection criteria

This narrative review considered peer-reviewed research studies, systematic reviews, and major experimental reports focusing on the application of AI in Alzheimer’s disease diagnosis, prognosis, and therapy development. The inclusion criteria were:

Study types: Human-based studies, including both observational and interventional designs, as well as major systematic reviews. Preclinical animal studies were considered only if they directly contributed to AI algorithm development or translational validation in AD models.

AI modalities: Studies employing machine learning, deep learning, natural language processing, reinforcement learning and hybrid AI systems. Traditional statistical models without AI elements were excluded.

Clinical focus: Research focused on Alzheimer’s disease, including typical AD, mild cognitive impairment and atypical AD subtypes (e.g., posterior cortical atrophy, logopenic variant PPA), if AI was used to differentiate or diagnose.

Imaging and data modalities: Studies using neuroimaging (MRI, fMRI, PET, EEG), omics datasets (genomic, transcriptomic, proteomic), electronic health records (EHRs), and speech or behavioral data for AI modeling were included.

Language and access: Only English-language publications with accessible full texts from peer-reviewed journals and conference proceedings published between 2000 and 2024 were considered.

## 3 Search strategy and literature selection

Although not conducted as a systematic review, a structured search strategy was used to identify relevant literature. The following academic databases were consulted:

- PubMed/MEDLINE
- IEEE Xplore
- Scopus
- Web of Science
- Google Scholar (for landmark or gray literature)

The search timeframe covered publications from January 2000 to April 2024. Combinations of Medical Subject Headings (MeSH) and keywords were used, including:

“Alzheimer’s disease,” “mild cognitive impairment,” “dementia,” “artificial intelligence,” “machine learning,” “deep learning,” “natural language processing,” “MRI,” “PET,” “biomarker,” “clinical trial,” “drug discovery,” “cognitive rehabilitation,” “prediction,” “diagnosis”

Manual screening of titles and abstracts was performed to assess relevance. Studies were excluded that:

- Focused on unrelated neurological disorders,
- Used traditional models without AI integration,
- Lacked methodological clarity or clinical applicability.

This work does not follow PRISMA guidelines, as it was designed as a narrative literature review to explore emerging and diverse applications of AI in Alzheimer’s disease, rather than to systematically synthesize evidence or perform meta-analysis.

## 4 Epidemiology in China and the world

**FIGURE 1:**
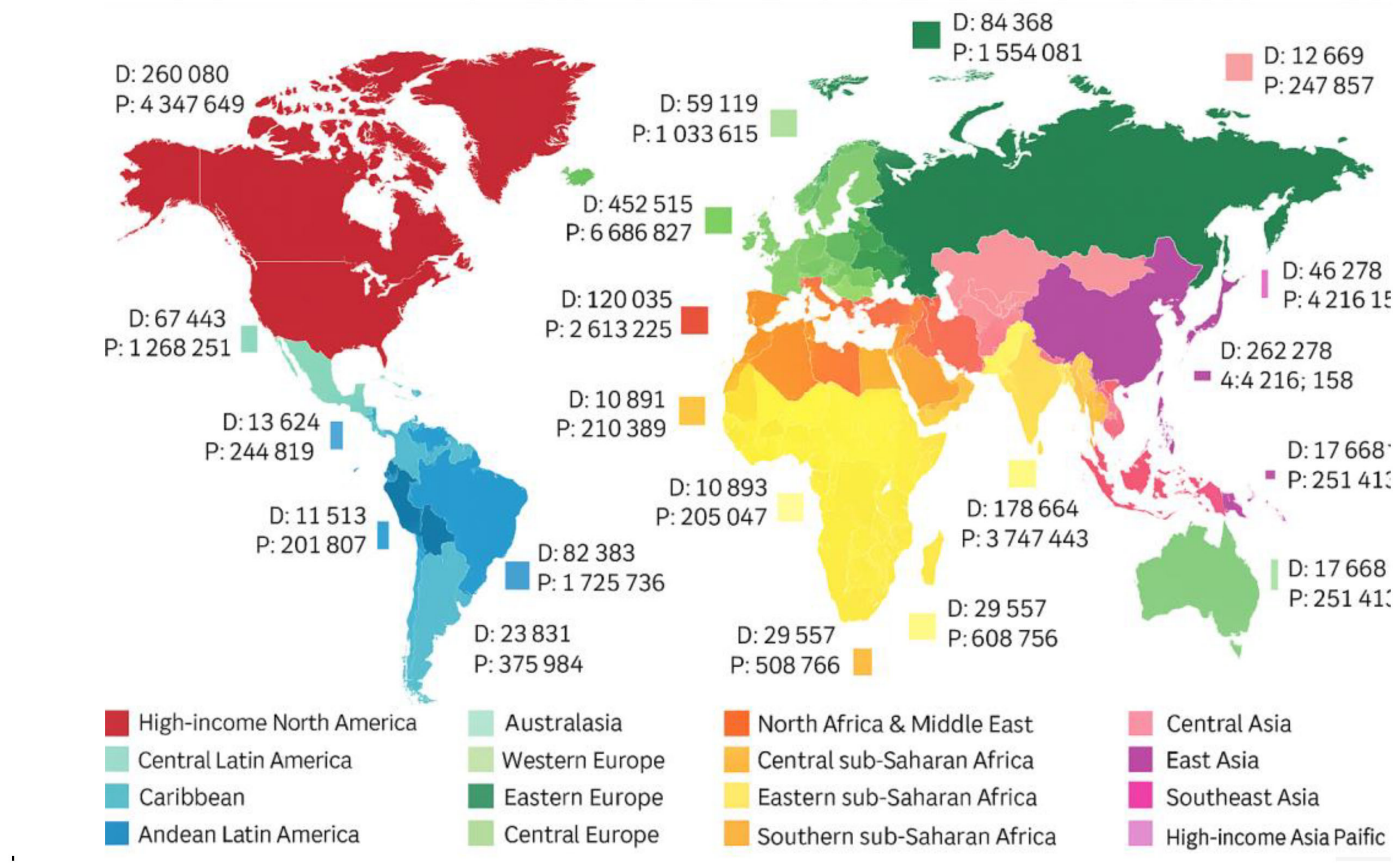
Global prevalence (P) and mortality (D) estimates for Alzheimer’s disease and other dementias. The figure illustrates the increasing burden of AD across various world regions based on epidemiological data compiled by Cano et al. (2021).

Currently, AD affects over 50 million people globally, and based on estimates, this number is expected to triple by 2050 due to an increase in the aging population (Maturi et al., 2022). Figure 1 illustrated the global prevalence and mortality associated with AD. According to a report from Beijing in 2023, more than 15 million Chinese aged 60 or more had dementia, with over 10 million individuals diagnosed with AD. It is postulated that the rapid aging of the Chinese population, combined with increasing life expectancy, might be contributing to this growing public health concern (Graham et al., 2020). Considering the estimates that by 2040, 28% of the Chinese population will be older, China will have the largest population of AD patients in the world (Mirkin and Albensi, 2023). This increase poses substantial challenges for the healthcare system, caregivers, and society at large, demanding urgent action to improve molecular understanding, diagnosis, treatment, and especially care for those affected (Farid et al., 2020).

## 5 Impact on society

**FIGURE 2:**
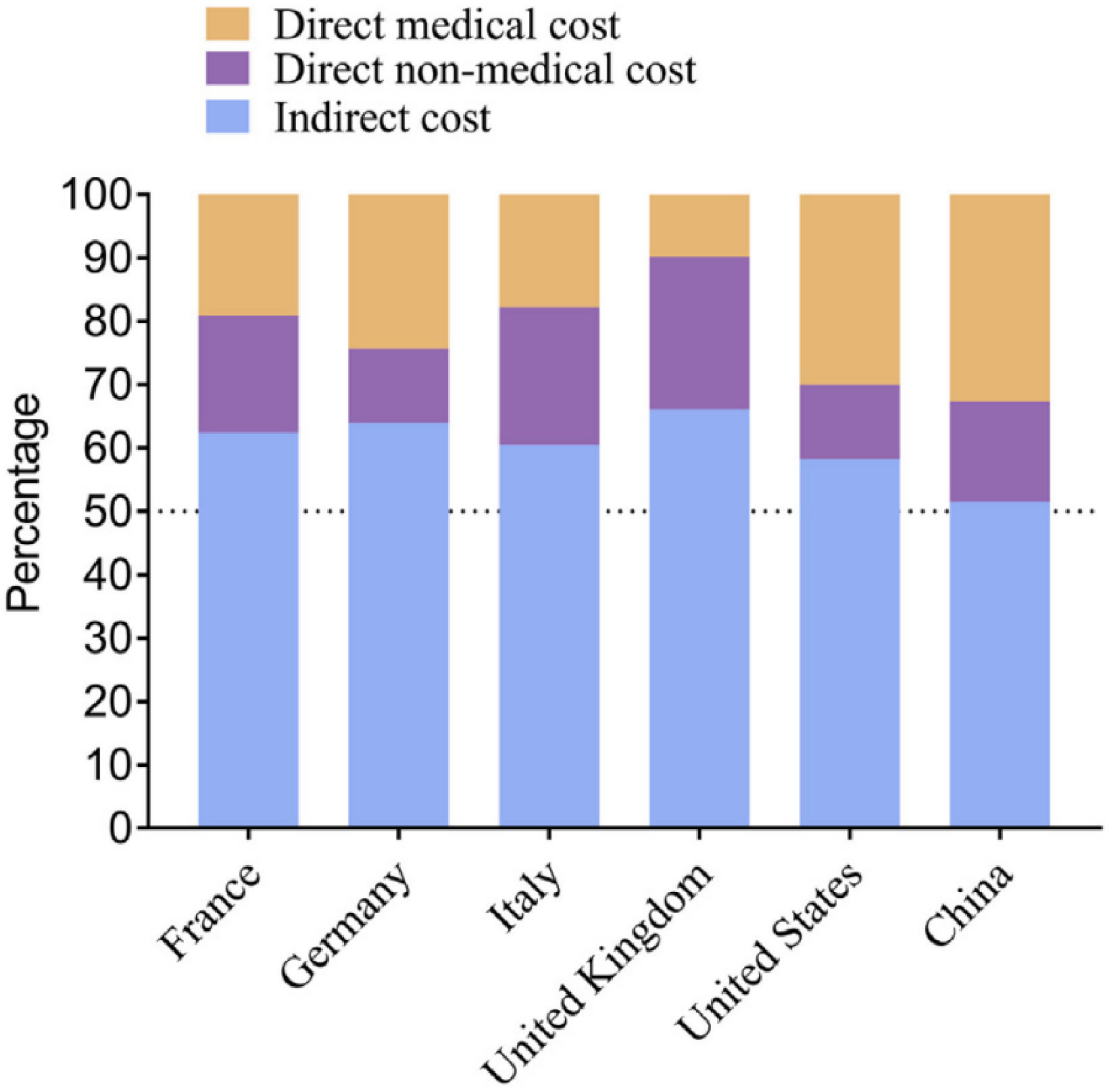
Estimated economic burden of Alzheimer’s disease in selected developed countries, including direct healthcare costs and long-term care expenditures. Adapted from Jia et al. (2018).

Alzheimer’s disease has a profound impact on society, where it affects not only individuals diagnosed but also their families and caregivers. This impact often comes with a substantial emotional and financial burden of caring for someone with AD. This burden has been reported to affect caregivers who experience significant stress and physical health issues (Logan et al., 2021). Apart from being the fifth leading cause of global mortality in the aged (> 65) population, the healthcare cost of managing AD is staggering, encompassing direct medical expenses, long-term care costs, and lost productivity. In 2020, the healthcare cost of AD was estimated to be $305 billion. However, with the rise in the aging population, this number is expected to be over $1 trillion in the coming decades (Ienca et al., 2017). Figure 2 estimates the economic burden of AD on major developed countries [10]. Burdens like these require substantial resources from healthcare systems to manage the ever-growing number of AD patients.

## 6 Relevance of AI in healthcare

Much like the revolutionary contributions that AI is making in almost every sector of our complex life, the utilization of AI has been promising in transforming healthcare, especially in enhancing diagnostics, effective treatment planning, and improving patient care. Considering the rise of AI in healthcare, it has already been estimated that by 2026, the application of AI is expected to reduce annual United States healthcare costs by $150 billion (Bazarbekov et al., 2024; Mittal and Hazari, 2023). In the context of AD, we are counting on the ability of AI technologies to improve early detection, predict disease progression, and identify potential therapeutic targets (Cheng and Cummings, 2022). Machine learning algorithms have the potential to analyze large and multiple volumes of medical data, including imaging, genetic, and clinical information, to uncover patterns indicative of AD. By harnessing AI-powered tools, clinicians can make more accurate and timely diagnoses, develop personalized treatment plans, and subsequently monitor disease progression. AI offers major advantages in identifying novel drug targets and optimizing clinical trial design, accelerating drug discovery (Subramanian et al., 2024). With these applications, the integration of AI in healthcare certainly has the potential to revolutionize the management of AD, which includes the improvement in patient outcomes and reducing its rising burden on healthcare systems. Table 1 provides a summary of some recent studies that used AI in the healthcare and management of AD.

**TABLE 1:** Summary of some recent studies that used artificial intelligence (AI) in the healthcare and management of Alzheimer’s disease (AD).

Study	AI tool used	Aim of the study	Main outcome and results	References
Machine learning methods for predicting progression from mild cognitive impairment to Alzheimer’s disease dementia: a systematic review	Support Vector Machine, Convolutional Neural Networks	Predict whether patients with mild cognitive impairment might develop Alzheimer’s disease dementia or remain stable	SVM achieved a mean accuracy of 75.4%, while CNN achieved a higher mean accuracy of 78.5%. Combining MRI and PET data improved classification accuracy	Grueso and Viejo-Sobera, 2021
Deep learning in Alzheimer’s disease: diagnostic classification and prognostic prediction using neuroimaging data	Convolutional Neural Network, Recurrent Neural Network, Stacked Auto-Encoder	Early detection and automated classification of Alzheimer’s disease using neuroimaging data	Deep learning approaches yielded accuracies up to 96.0% for AD classification and 84.2% for MCI conversion prediction	Jo et al., 2019
Machine learning and novel biomarkers for the diagnosis of Alzheimer’s disease	Support Vector Machine, Logistic Regression, Random Forest, NaÃ^–^ve Bayes	Investigate machine learning and novel biomarkers for the diagnosis of AD	Machine learning with novel biomarkers increased sensitivity and specificity in AD diagnosis	Chang et al., 2021
Machine learning for comprehensive forecasting of Alzheimer’s disease progression	Conditional Restricted Boltzmann Machine	Simulate detailed patient trajectories for personalized forecasting of disease progression	CRBM accurately reflected means, standard deviations, and correlations of clinical variables over time	Fisher et al., 2018
Machine learning approaches and applications in genome wide association study for Alzheimer’s disease: A systematic review	Artificial Neural Networks, Boosting, Random Forests	Examine the use of machine learning for the prediction of Alzheimer’s disease based on genetic data	Machine learning methods performed with AUC ranging from 0.59 to 0.98	Alatrany et al., 2022
Multivariate data analysis and machine learning in Alzheimer’s disease with a focus on structural magnetic resonance imaging	Various classifiers, Feature extraction algorithms	Review studies using machine learning and multivariate analysis in AD research, focusing on structural MRI	Wide variety of methods and outcomes; influential factors include classifiers, feature extraction, and validation methods	Falahati et al., 2014
Early-stage Alzheimer’s disease prediction using machine learning models	Decision Tree, Random Forest, Support Vector Machine, Gradient Boosting, Voting Classifiers	Identify the best parameters for Alzheimer’s disease prediction	Best validation average accuracy of 83% on test data of AD	(Kavitha et al., 2022)
Machine learning algorithms and statistical approaches for Alzheimer’s disease analysis based on resting-state EEG recordings: a systematic review	Support Vector Machines	Summarize recent publications on AD detection and correlation of EEG features with AD progression	Promising conclusions for future studies; deep learning techniques not yet applied on large EEG datasets	Tzimourta et al., 2021
Transfer learning with intelligent training data selection for prediction of Alzheimer’s disease	VGG architecture with pre-trained weights	Improve AD detection from MRI data using transfer learning and intelligent training data selection	Achieved state-of-the-art performance with smaller training size; 4% and 7% increase in accuracy for AD vs. MCI and MCI vs. NC, respectively	Khan et al., 2019
An improved multi-modal based machine learning approach for the prognosis of Alzheimer’s disease	Random Forest	Present an automated classification system for AD prognosis using MRI data	Random Forest classifier showed the highest output in classification accuracy	Khan and Zubair, 2022

## 7 AI technologies in Alzheimer’s disease research

### 7.1 Machine learning (ML) algorithms

Machine learning (ML) plays a key role in Alzheimer’s research by uncovering patterns in complex, high-dimensional datasets that may be overlooked by human analysis (Li et al., 2022). Three major types of ML are used: supervised, unsupervised, and reinforcement learning. Supervised learning trains algorithms on labeled data and is widely applied to early diagnosis and disease progression prediction, particularly using neuroimaging datasets (Verma et al., 2022). Unsupervised learning explores unlabeled data to identify novel biomarkers or patient subtypes (Patel, 2022). Reinforcement learning, though less common, is increasingly used to optimize treatment strategies based on patient feedback loops (Verma et al., 2022). Whereas unsupervised learning deals with unlabeled data to identify hidden patterns without prior knowledge of the outcomes. In the case of AD research, unsupervised learning algorithms can be used to discover novel biomarkers or for the purpose of classifying patients into different subtypes based on their disease characteristics (Patel, 2022). However, reinforcement learning has less common utilization in AD research; nevertheless, it is gaining applications in optimizing treatment strategies by learning from interactions within a given environment.

**FIGURE 3:**
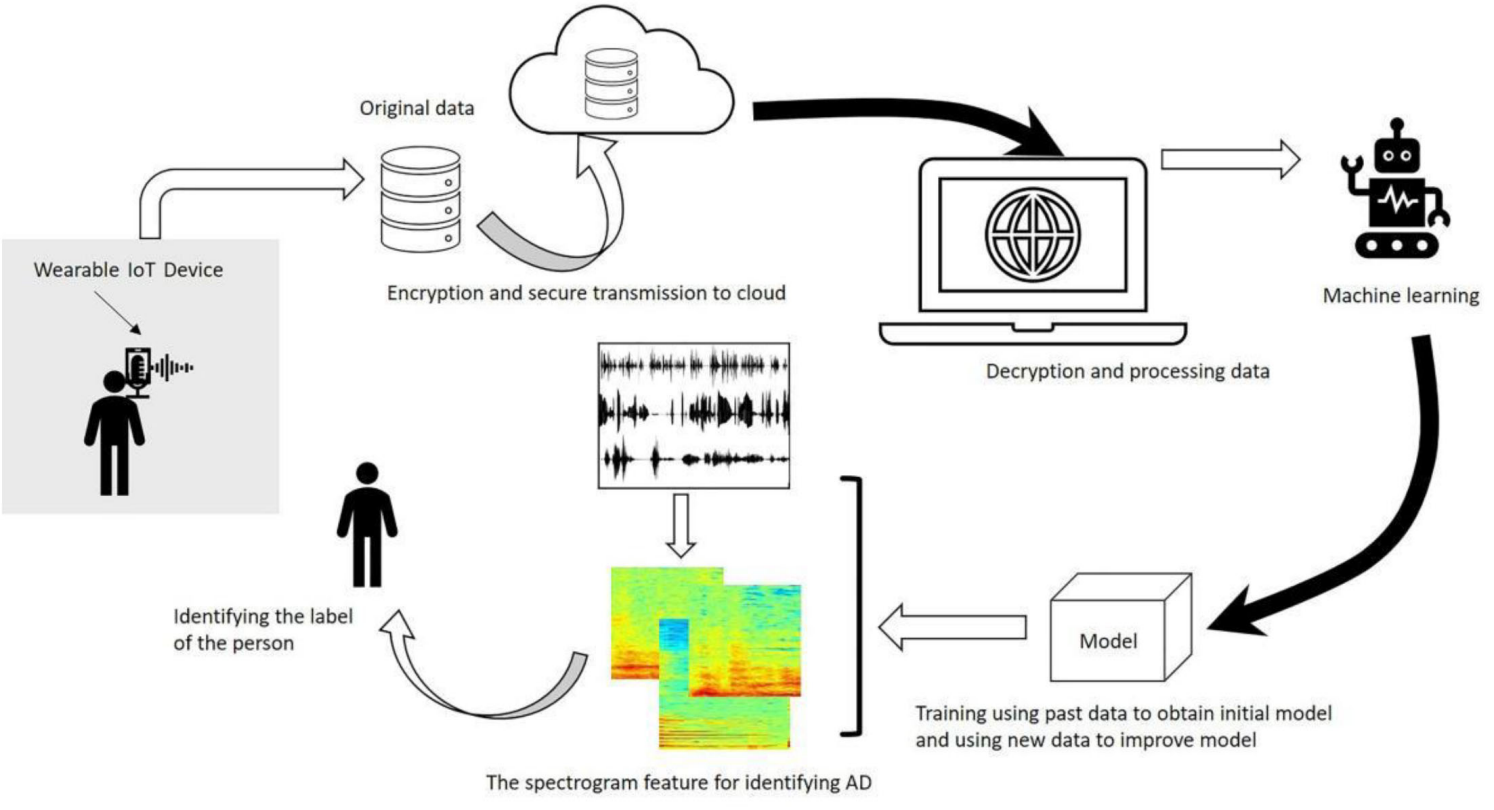
Workflow diagram illustrating the application of machine learning algorithms in Alzheimer’s disease diagnosis. The architecture includes data preprocessing, feature selection, model training (e.g., SVM, RF), and validation using neuroimaging datasets. Adapted from Liu et al. (2020).

In conclusion, the examples of ML applications in AD majorly include the development of predictive models that can accurately forecast disease progression by analyzing the data from the clinical, genetic, and lifestyle of the patients. Through these models, clinicians can receive assistance in the identification of individuals at high risk for developing AD, thereby enabling early intervention (Ghosh et al., 2021). Recently, ML algorithms have been developed and used to analyze speech patterns and cognitive test results that are aimed at providing non-invasive and cost-effective diagnostic approaches (Xie et al., 2020). Figure 3 adapted from Liu et al. (2020), highlights the architectural workflow of diagnosing AD using machine learning methods (Seo et al., 2022).

**FIGURE 4:**
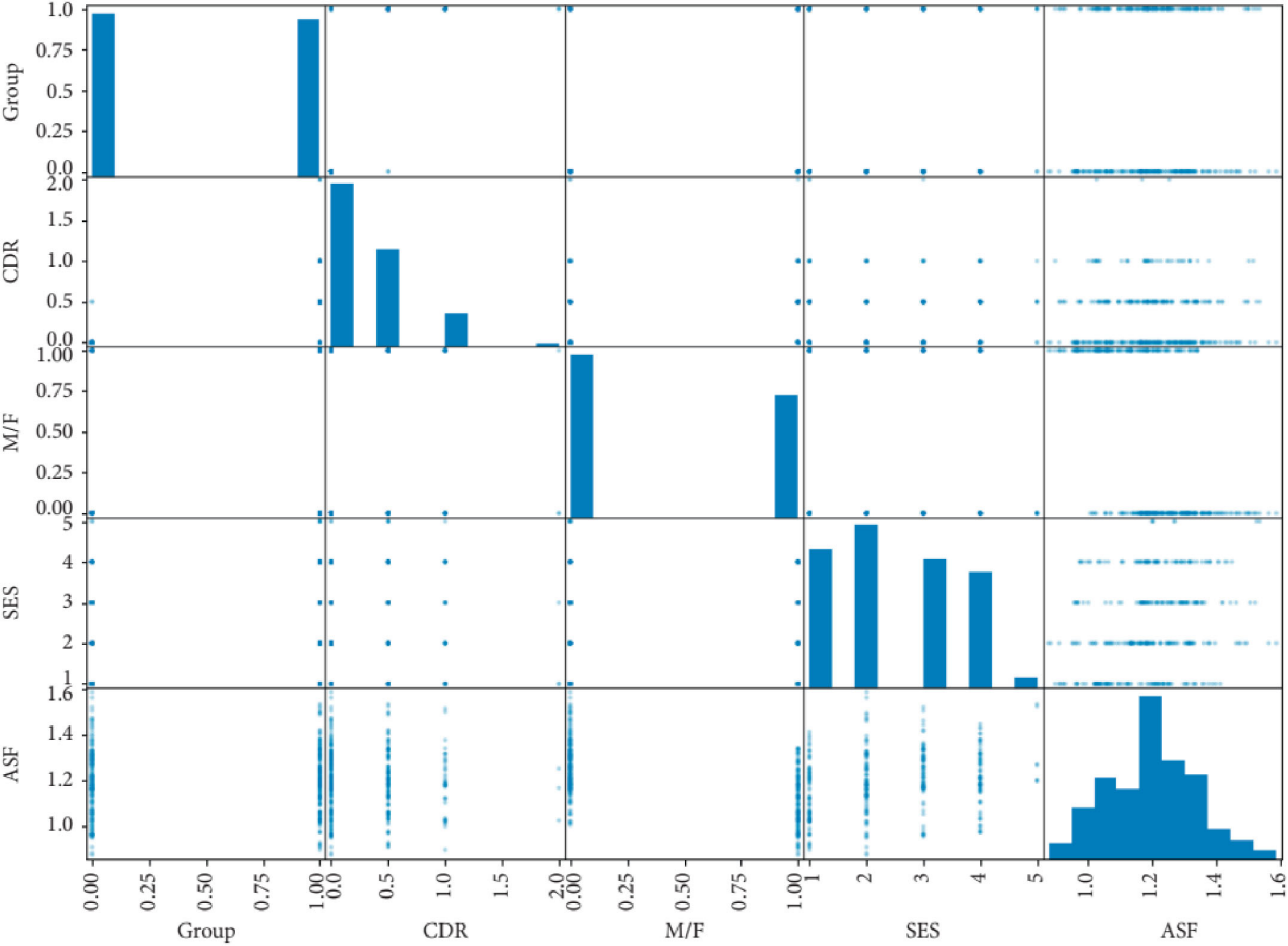
Correlation matrix of clinical variables in an Alzheimer’s disease dataset, used to train machine learning models including support vector machines and logistic regression. Adapted from Bari Antor et al. (2021).

Predicting the onset of Alzheimer‘s disease is the system‘s primary objective. An adult patient‘s risk of developing Alzheimer‘s disease or dementia may be estimated using data from the “MRI and Alzheimer’s” dataset, which is part of the Open Access Series of Imaging Studies (OASIS) initiative. The missing values have been filled in and the dataset has been shown. Some superfluous characteristics have been removed from the data as part of the preprocessing. The e-values were standardized so that the ML models could use them with ease. Support vector machines, logistic regression, decision trees, and random forest models have all been trained on this dataset (Figure 4). Accuracy, recall, area under the curve, and confusion matrix are the measures that have been used for assessment. Every model that has been generated has been fine-tuned using the grid search approach in order to enhance the system output. Using SVM, the system achieved the best result for this specific dataset. The random forest classifier, which is a more complicated model, suffered from an overfitting problem. The SVM model outperformed all other models when it came time for deployment. Adding additional ML models like AdaBoost, KNN, Majority Voting, and Bagging to the system models in the future might be a great way to enhance them. (will) make the system more dependable and efficient (Bari Antor et al., 2021).

(a) Deep learning (DL)

Deep learning (DL) is a derivative of ML that uses different neural networks with multiple layers to model and simplify complex data. The data from the multimodal studies of AD generated vast information like demographics, cognitive scores, demographics, neuropathology vital signs, symptoms, medical history, neuropsychological battery, lab tests, etc., This data can be used to generate models like clinical decision support systems (CDSS) for AD that aim to bring early diagnostics and monitoring in the primary or patient routine care (Seo et al., 2022). For that purpose, in AD research, Convolutional Neural Networks (CNNs) are particularly effective for analyzing imaging data. CNNs are pretty efficient at identifying spatial hierarchies in images, and they can process brain scans like MRI and PET images. By learning to recognize patterns associated with AD, CNNs can, therefore, be utilized to detect early structural and functional brain changes, facilitating early diagnosis and monitoring disease progression (Vrahatis et al., 2023).

Recurrent Neural Networks (RNNs) are another type of DL algorithm that can analyze temporal data, and this application can be significant in predicting the progression of AD. Since RNNs are designed to handle sequential data, they are helpful in analyzing time-series data such as longitudinal patient records and cognitive test results. In AD research, RNNs can track changes in mental function over time, providing insights into disease progression and helping predict future decline (Graham et al., 2020). However, in such applications, missing data from longitudinal studies often causes errors in the prediction that have been challenging the success of RNN models in this application of DL. To counteract this, modern-day models like mimimalRNN are using strategies like “forward filling,” “linear filling,” and “model filling” to fill the missing data gaps using extrapolation of available data (Zhao et al., 2023).

**FIGURE 5:**
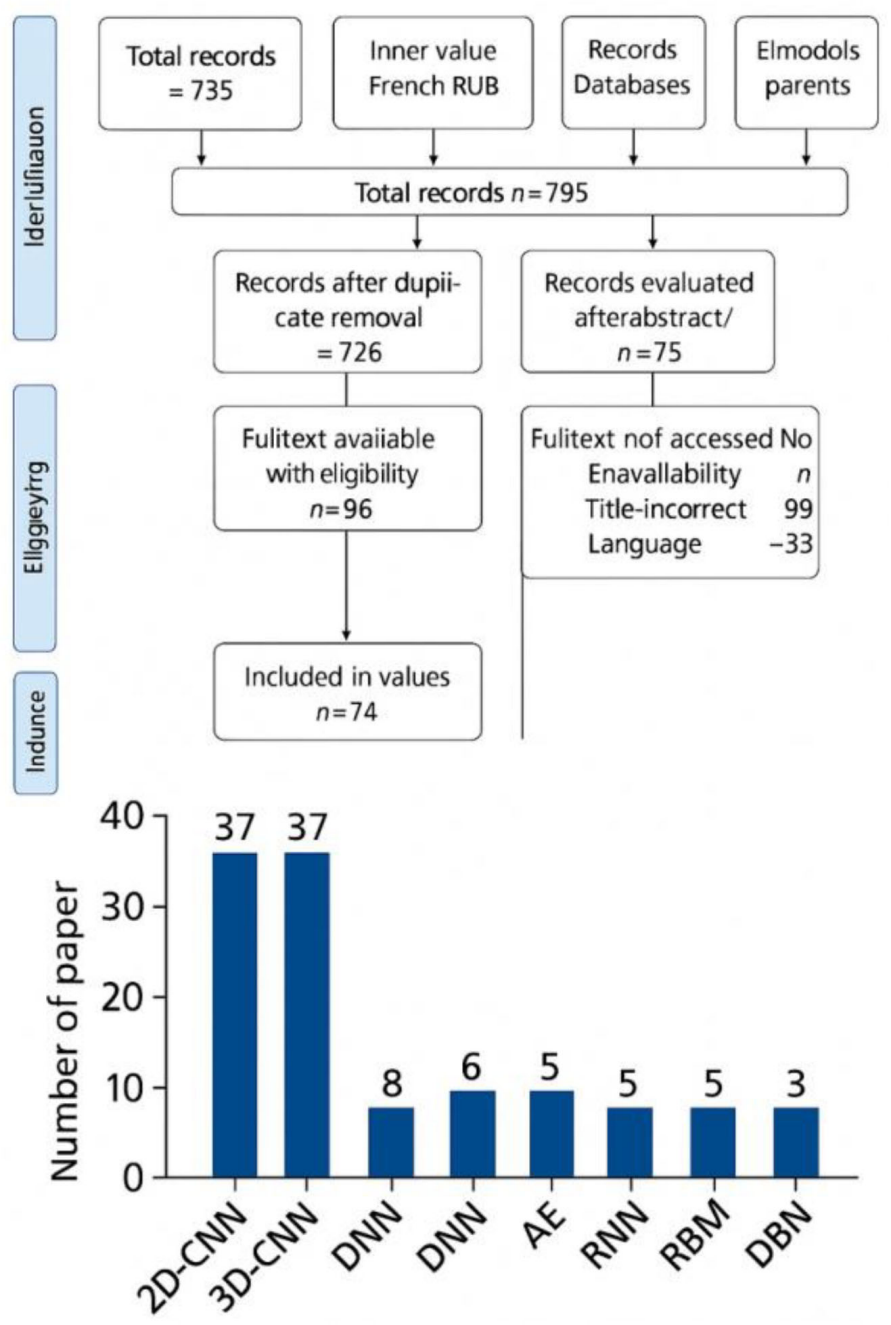
Overview of deep learning models applied to neuroimaging data for early diagnosis of Alzheimer’s disease. The figure highlights classification between normal cognition (NC), mild cognitive impairment (MCI), and Alzheimer’s disease (AD) using CNN-based architectures. Adapted from Fathi et al. (2022).

Early detection of AD is crucial for halting its progression and improving the quality of life for both patients and their families. Given the difficulty physician’s face in diagnosing early stages of AD, such as normal cognition (NC), mild cognitive impairment (MCI), and early MCI (eMCI), deep learning systems have attracted significant attention. Fathi et al. (2022) systematically reviewed how deep learning methods, particularly CNN-based models, have been applied to neuroimaging data (MRI, fMRI, PET) for early AD diagnosis (Figure 5). Their focus was on more challenging and clinically valuable classifications, like NC/MCI and NC/eMCI, which are critical for early detection. Promising results had reported using CNNs, particularly when employing 3D structures on large datasets. Models like DenseNet, ResNet, and VGG consistently perform well, though newer architectures, like CapsNet, still require further investigation. While MRI and PET offer superior performance to fMRI, multimodal approaches, despite their potential, are more expensive and time-consuming. Findings suggested that deep learning has strong potential to enhance computer-aided diagnosis (CAD) systems, aiding physicians in early AD detection and offering screening tools for at-risk populations. However, further research is necessary for optimizing feature extraction strategies and improving model generalization (Fathi et al., 2022).

(i) Feed-forward DNN for AD diagnosis

**FIGURE 6:**
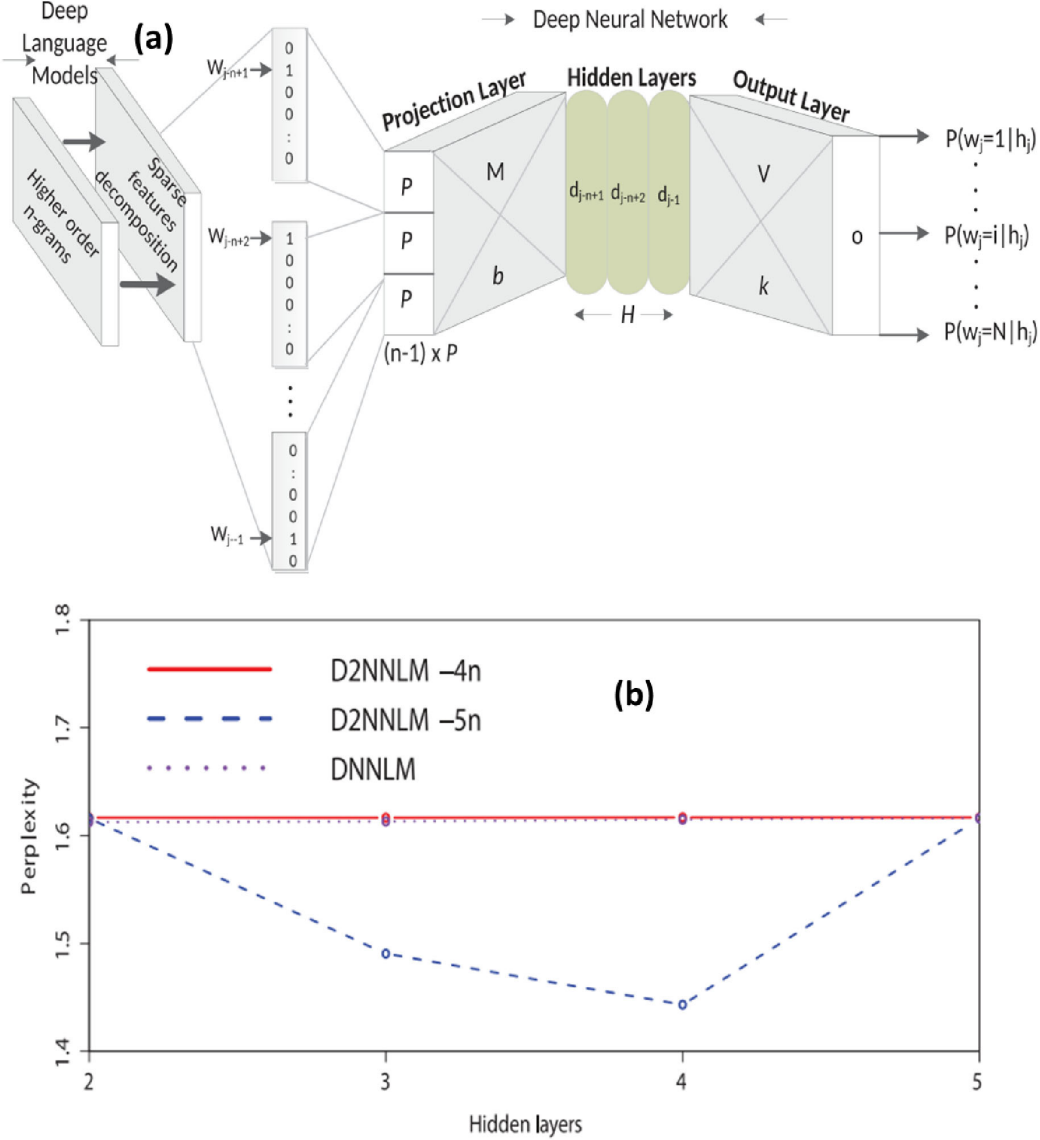
**(a)** Architecture of the Deep Deep Neural Language Model (D2NNLM) for predicting Alzheimer’s disease progression based on linguistic features. **(b)** Comparison of model perplexity across different hidden layers for D2NNLM and baseline DNNLM on AD-type dementia datasets. Adapted from Orimaye et al. (2018).

Fruehwirt et al. (2018) created a model using Bayesian Deep Neural Networks to predict the severity of Alzheimer’s disease using EEG data. Each layer of the proposed models has 100 units. In clinical neuroscience, the authors demonstrated that the proposed model effectively predicts disease severity (Fruehwirt et al., 2018). Orimaye et al. (2018) proposed a hybrid model combining DNN and D2NNLM for Alzheimer‘s disease prediction. The studies conducted in the research demonstrated that the proposed model well forecasts the advancement of moderate cognitive impairment to Alzheimer‘s disease (Figures 6a, b). On both datasets, D2NNLM-4n and D2NNLM-5n outperform DNNLM and NNLM in terms of percentage mistakes and, on average, complexity. In contrast, we found that D2NNLM-5n performs worse than D2NNLM-4n. We found that D2NNLM-4n produced an ideal model with greater generalization error, while D2NNLM-5n produced an excellent language model with reduced perplexity, even if D2NNLM-5n’s performance was still superior to DNNLM and NNLM. Regarding the robustness of our models with less hidden layers, we saw a minor performance difference on the AD-type dementia dataset. Specifically, at the third and fifth hidden levels, D2NNLM-4n produced a lower percentage error. Hidden layer 3 was where the D2NNLM-5n achieved its lowest percentage error, in contrast to the MCI dataset. In contrast, hidden layers 3, 4, and 5 of the D2NNLM-4n demonstrated more robust and consistent performance. These findings probably point to the distinction between Alzheimer’s disease (AD) type dementia research issues and mild cognitive impairment (MCI) (Bacci, 2017; Bucholc et al., 2023; Cano et al., 2021). Patients with mild cognitive impairment (MCI) often have identical language traits to healthy control persons, suggesting that much longer word dependencies may be necessary to capture their linguistic deficiencies. However, the D2NNLM-4n may readily capture linguistic deficiencies in AD-type dementia patients without overstretching the language model. On average, D2NNLM-4n and D2NNLM-5n outperformed DNNLM across all five hidden layers, even though the percentage errors for AD-type dementia were greater than for MCI (Orimaye et al., 2018).

Ning et al. (2018) developed a model for subject classification into AD and CN categories via a neural network. The model predicts that individuals with moderate cognitive impairment will advance to Alzheimer’s disease. The model received MRI and genetic data. Upon comparing the proposed model to logistic regression (LR), the authors determined that the former exhibited superior performance (Ning et al., 2018).

Park et al. (2020) introduced a DNN-based model to predict the progression of Alzheimer’s disease, using data on DNA methylation and integrated gene expressions as inputs. The authors demonstrated that integrated data yields more accurate models than single-modal data. The proposed model outperformed the current state-of-the-art in machine learning. The authors used the Bayesian technique to determine the optimal model parameters. A deep neural network with eight hidden layers, each containing 306 nodes, with a learning rate of 0.02 and a dropout rate of 0.85, demonstrated superior performance relative to other models (Park et al., 2020). To categorize individuals into No-Dementia (ND), Mild Cognitive Impairment (MCI), and AD Benyoussef et al. (2019) proposed a hybrid model integrating KNN (K-Nearest Neighbor) and DNN, using MRI data. The proposed model used KNN to assist DNN in differentiating between easily diagnosable and challenging-to-diagnose patients. Each hidden layer of the DNN had one hundred nodes. The experimental results demonstrated that the proposed model effectively differentiated among the many stages of Alzheimer’s disease (Benyoussef et al., 2019).

(ii) Utilization of convolutional neural networks in Alzheimer’s disease diagnosis

This study used CNN to diagnose AD. Suk and Shen (2016) proposed a hybrid model for Alzheimer’s disease diagnosis using both Sparse Regression Networks and Convolutional Neural Networks. The model used many Sparse Regression Networks to provide various representations at the target level. Subsequently, CNN effectively identified the output label by synthesizing these target-level representations (Suk and Shen, 2016). To classify individuals into three groups—AD, MCI, and HC—utilizing structural MRI data, Billones et al. (2016) adapted the 16-layer VGGNet. The authors achieved precise classifications based on the study’s tests. The authors assert that this was achieved without MR image segmentation. Recent studies have shown encouraging outcomes in the diagnosis of AD and Mild Cognitive Impairment (MCI) using structural Magnetic Resonance Imaging (MRI) images. This research proposes using a Convolutional Neural Network (CNN) for the identification of AD and Mild Cognitive Impairment (MCI). Specifically, we used the 16-layer VGGNet for the tri-class classification of Alzheimer’s disease (AD), Mild Cognitive Impairment (MCI), and Healthy Controls (HC) using the Alzheimer’s disease Neuroimaging Initiative (ADNI) dataset, attaining an overall accuracy of 91.85% and surpassing other classifiers from previous research (Billones et al., 2016).

**FIGURE 7:**
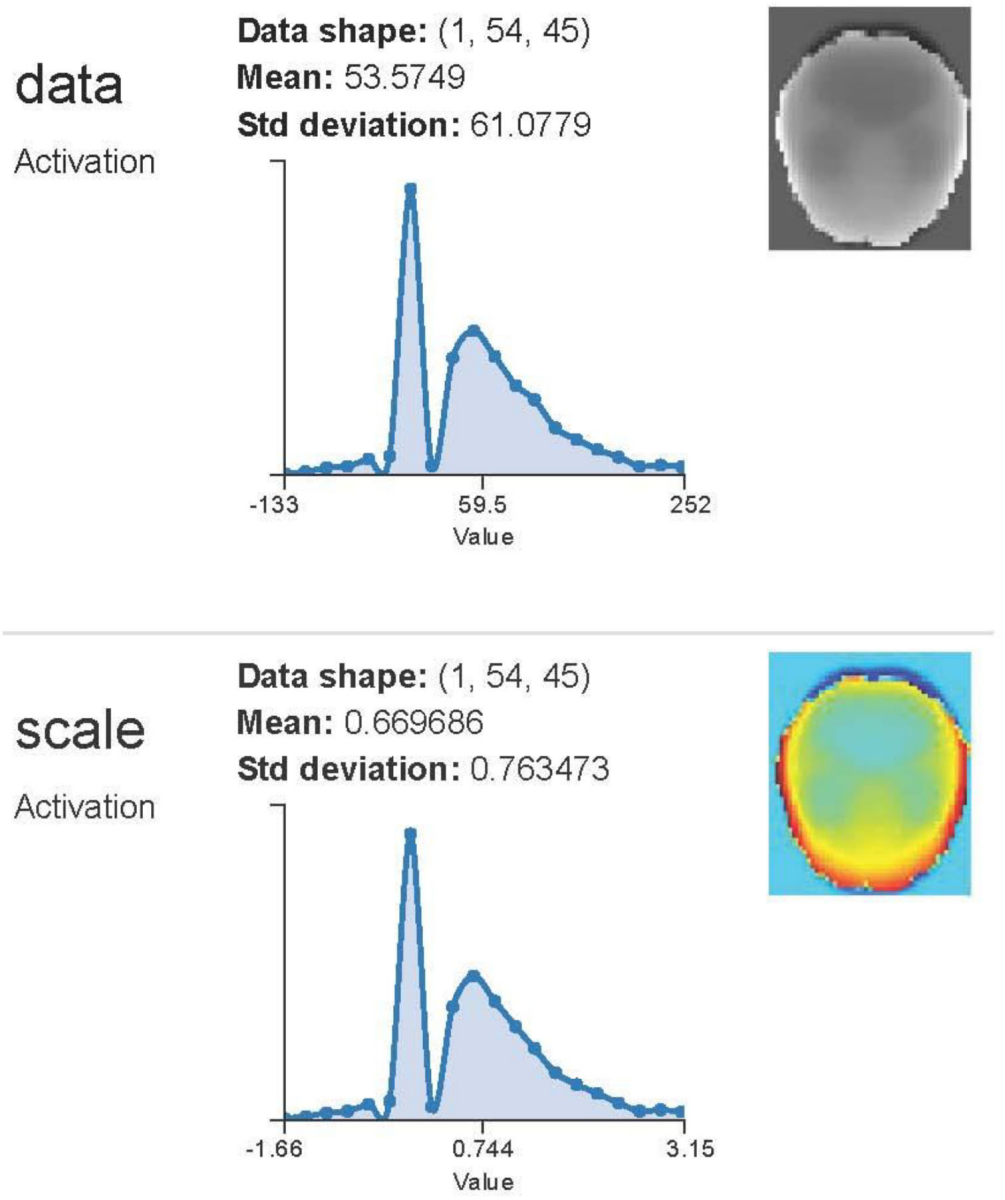
Visualization of feature maps from convolutional neural networks applied to structural and functional MRI data for Alzheimer’s classification using LeNet and GoogleNet architectures. Adapted from Sarraf et al. (2016).

Sarraf and Tofighi (2016) used LeNet architecture to differentiate between Alzheimer’s disease and healthy subjects via functional MRI. The researchers concluded that CNN’s scale- and shift-invariant characteristics provide significant promise in medical imaging (Sarraf and Tofighi, 2016). Sarraf and Tofighi used structural MR images to differentiate Alzheimer’s disease patients from healthy individuals with the LeNet design. Figure 7 showed the final filters of several layers. CNN is an effective feature extractor because to its convolutional layers, which can extract high-level information from pictures. This deep learning approach serves as a robust classifier due to its intricate network design. Our present strategy, which employs comprehensive preprocessing techniques followed by CNN classification, enhanced the accuracy of Alzheimer’s disease data classification from 84% using Support Vector Machine (SVM). Nonetheless, deep learning systems present many challenges, including significant algorithmic complexity and costly infrastructure requirements (Sarraf and Tofighi, 2016).

The study had an accuracy rate of 98.84%. Sarraf and Tofighi (2016) performed a supplementary study using functional and structural MR images to detect Alzheimer’s disease with LeNet and GoogleNet architectures. The trials conducted in the research demonstrated that these structures surpassed the most advanced techniques for identifying Alzheimer’s disease (Sarraf et al., 2016).

Gunawardena et al. (2017) used structural MRI to create a CNN-based methodology for the early identification of Alzheimer’s disease. The study indicated that the CNN model outperformed the SVM in the evaluation of the proposed method’s efficacy. In further experiments, the researchers want to include two more MRI perspectives—axial and sagittal—alongside the coronal picture used in this study (Gunawardena et al., 2017). Basaia et al. (2019) developed a CNN-based model for Alzheimer’s disease diagnosis using structural MR images. To mitigate overfitting and enhance computational efficiency, the study used data augmentation and transfer learning techniques. The authors said that this study overcomes the limitations of prior research by broadening its coverage beyond single-center datasets (Basaia et al., 2019).

(b) Natural Language Processing (NLP)

Since the majority of medical data is unstructured and in the form of electronic health records (eHRs), substantial input is required to extract the relevant information. NLP is a branch of AI that focuses on understanding human language and converting it into machine language that computers can utilize to lay meaningful interpretations. NLP has been recently employed to understand the clinical notes of the patients (Hofmann et al., 1996a). Similarly, in AD research, NLP techniques have been utilized to analyze patient records and their clinical notes (Patel et al., 2021). By extracting relevant information from unstructured text, NLP algorithms can identify patterns and trends that might indicate early signs of cognitive decline (Shanmugavadivel et al., 2023). For example, changes in language use and complexity in medical records can serve as early indicators of AD.

Additionally, with the advancement in the development of algorithms, NLP has also been used to analyze speech and language patterns directly (Aditya Shastry and Sanjay, 2024). Studies have shown that subtle changes in speech, such as reduced vocabulary diversity and increased pause frequency, can be indicators of early markers of cognitive impairment. NLP can analyze large speech datasets to detect early signs of cognitive decline and track disease progression through changes in language patterns over time (Fabrizio et al., 2021).

This study aims to assess how well cognitive assessments and biomarkers are documented in EHRs, which are real-world endpoints; second, it will use natural language processing (NLP) techniques to identify, extract, and standardize cognitive tests from clinical narratives into AD/ADRD severity levels; and third, it will assess how well EHRs document cognitive assessments and biomarkers. For the purpose of extracting biomarkers and cognitive tests from clinical narratives in EHRs of patients with AD and AD-related dementias, we built a rule-based natural language processing pipeline (Figure 9). We used patient-level data consolidation and cognitive test score standardization based on cutoffs developed from relevant literature and the knowledge of AD/ADRD physicians to classify patients into severity groups. From the UF Health system, we identified a sample of 48,912 people with AD/ADRD and identified seven metrics often documented in our data: six cognitive exams and one biomarker. Over all seven tests, our NLP pipeline achieved an F1-score of 0.9059. Out of the six cognitive tests, we were able to classify four test scores into severity levels and identify patient demographics according to their test results. Other features related to the availability of their records in EHRs were also uncovered. The results of this study validate the efficacy of our natural language processing pipelines in extracting cognitive evaluations and biomarkers of Alzheimer’s disease and Alzheimer’s disease Related Disorders (ADRD). Electronic health records may not capture all cognitive tests and biomarkers, but real-world data is still useful for studying dementias associated with Alzheimer’s disease and other forms of the illness. On the other hand, EHRs also need a methodical approach to recording cognitive tests and biomarkers (Chen et al., 2023).

(c) AI in AD

The use of AI may facilitate personalized medical treatment (Silva-Spínola et al., 2022). Utilizing AI to assess individual medical data may lead to diminished adverse effects and enhanced treatment success by selecting the most effective medications (Li et al., 2021). AI is used for the cognitive stimulation of patients (Jangwan et al., 2022). For example, AI has the capacity to create tailored brain augmentation technologies, which might assist in illness prevention. Researchers discovered that older persons exhibited a higher adherence to recommended prescription regimens after the use of chatbots and voice-activated assistants (Ruggiano et al., 2021). Artificial intelligence and machine learning-driven chatbots and virtual assistants may mitigate the emotional and social isolation associated with an Alzheimer’s disease diagnosis, while also offering caregivers educational tools and methods to remain involved (Vatansever et al., 2021).

The search for innovative Alzheimer’s disease therapies may potentially benefit from artificial intelligence. Artificial intelligence (AI) can analyze extensive medical and research data to identify potential innovative drugs that may cure the disease (Fang et al., 2022). A recent work exemplifies the application of AI to uncover potential therapeutic targets by detecting similar mitophagy markers of Alzheimer’s disease across several animal models (Xie et al., 2022). Furthermore, artificial intelligence may expedite medication development via the use of deep generative models. These models have the potential to transform the design, optimization, and synthesis of both macromolecules and small molecules. Creating molecules with pharmaceutical properties, diverse drug-like characteristics, enhanced bioavailability, and similar applications exemplify possible uses in this field (Zeng et al., 2022). A recent use of machine learning in drug development included studies examining many species to identify potential mitophagy inducers for the treatment of Alzheimer’s disease. The researchers identified two compounds capable of enhancing memory, reinstating glutamatergic and cholinergic functions, diminishing amyloid-1β and tau pathologies, and alleviating Alzheimer’s disease pathological characteristics in nematode and mouse models by a machine learning and cross-species screening methodology (Zeng et al., 2022).

(i) Employing artificial intelligence to monitor individuals with Alzheimer’s disease

Various non-invasive methods exist to monitor patients’ daily activities using AI, including their dietary intake, physical activity, and sleep patterns. AI may analyze video records using wearable or user-friendly devices (such as smartphones, smart watches, and tablets) to detect signs of anxiousness, repetitive behaviors, or other behavioral abnormalities that may signify a disease (Malche et al., 2022). Moreover, telemedicine enables doctors and caregivers to alleviate their workload while remotely monitoring patients and providing immediate support.

Individuals with Alzheimer’s disease may have their sleep habits monitored with artificial intelligence (Saliba et al., 2023). One study used algorithms that identified MCI with an accuracy over 80% with the use of sleep monitors (Khosroazad et al., 2023). AI can also detect sleep disorders and aid clinicians in prescribing the most appropriate treatment. Finally, it is important to acknowledge that AI may be beneficial in avoiding and monitoring falls in people with Alzheimer’s disease. Artificial intelligence may use a patient’s smartphone sensors to track their movements and alert medical experts and caregivers of any possible falls (Alqahtani et al., 2022).

(ii) Utilization of artificial intelligence to examine omics data for the investigation of the etiologies of Alzheimer’s disease

A new integrated multi-omics investigation of Alzheimer’s disease data revealed several and often intersecting metabolic pathways associated with the condition. Examples of such pathways include inflammation, oxidative stress, mitochondrial dysfunction, neurotransmitter synapses, vitamin deficiencies, and coagulation pathways (Kodam et al., 2023). The use of a network-based artificial intelligence system (AlzGPS) to integrate multi-omics data and identify high-confidence Alzheimer’s disease risk genes for potential therapy targets is another exemplary instance. This platform delivers medication repurposing outcomes using advanced network proximity methodologies for innovative insights, data on Alzheimer’s multi-omics datasets, genes, and pharmaceuticals, along with an interactive and user-friendly online interface with instructive network visualizations. It provides potential tool chemicals for Alzheimer’s disease research. Moreover, recent research indicates that the integration of AI with omics data may uncover new blood biomarkers, such as microRNAs, capable of predicting the early development of dementia, namely moderate cognitive impairment (MCI) (Zhou et al., 2021).

(iii) Artificial Intelligence for the optimization and design of clinical trials

The use of AI may enhance the identification and selection of clinical trial participants, as well as decrease the screen failure rate. Artificial intelligence (AI) can analyze extensive medical data with machine learning algorithms to identify trial participants who fulfill the inclusion requirements. Predictive metrics for amyloidosis, using MCI progression, MRI, family history, and other variables, have been developed by AI (Seo et al., 2022). The protein and MRI AD biomarker screening methodologies has the capacity to substantially reduce the probability of clinical trial failure. This exceptional use of AI has the capacity to expedite patient recruitment for trials, reduce unnecessary screenings, enhance trial efficiency by pinpointing optimal candidates, and more.

In 2020, synthetic PET images were generated for patients in the Alzheimer’s disease, mild cognitive impairment, and control groups as part of a study using predictive AI methodologies. The generative adversarial network, which underpins this and similar models, produces photorealistic PET images while concurrently teaching an artificial intelligence system to discriminate between authentic and counterfeit images (Kavitha et al., 2022). The model surpasses the classification model trained on synthetic data by 10%, achieving a classification accuracy of 71.45% for control or Alzheimer’s disease categorization (Islam and Zhang, 2020).

Clinical trial data management is an additional domain that might benefit from AI capabilities. Artificial intelligence (AI) may analyze patient data using machine learning (ML) algorithms to assess the efficacy of medications and the response to therapy. Besides aiding in the discovery of the most effective medications, these ML models may be used to formulate synergistic drug combinations that significantly improve treatment outcomes (Kuenzi et al., 2020).

**FIGURE 8:**
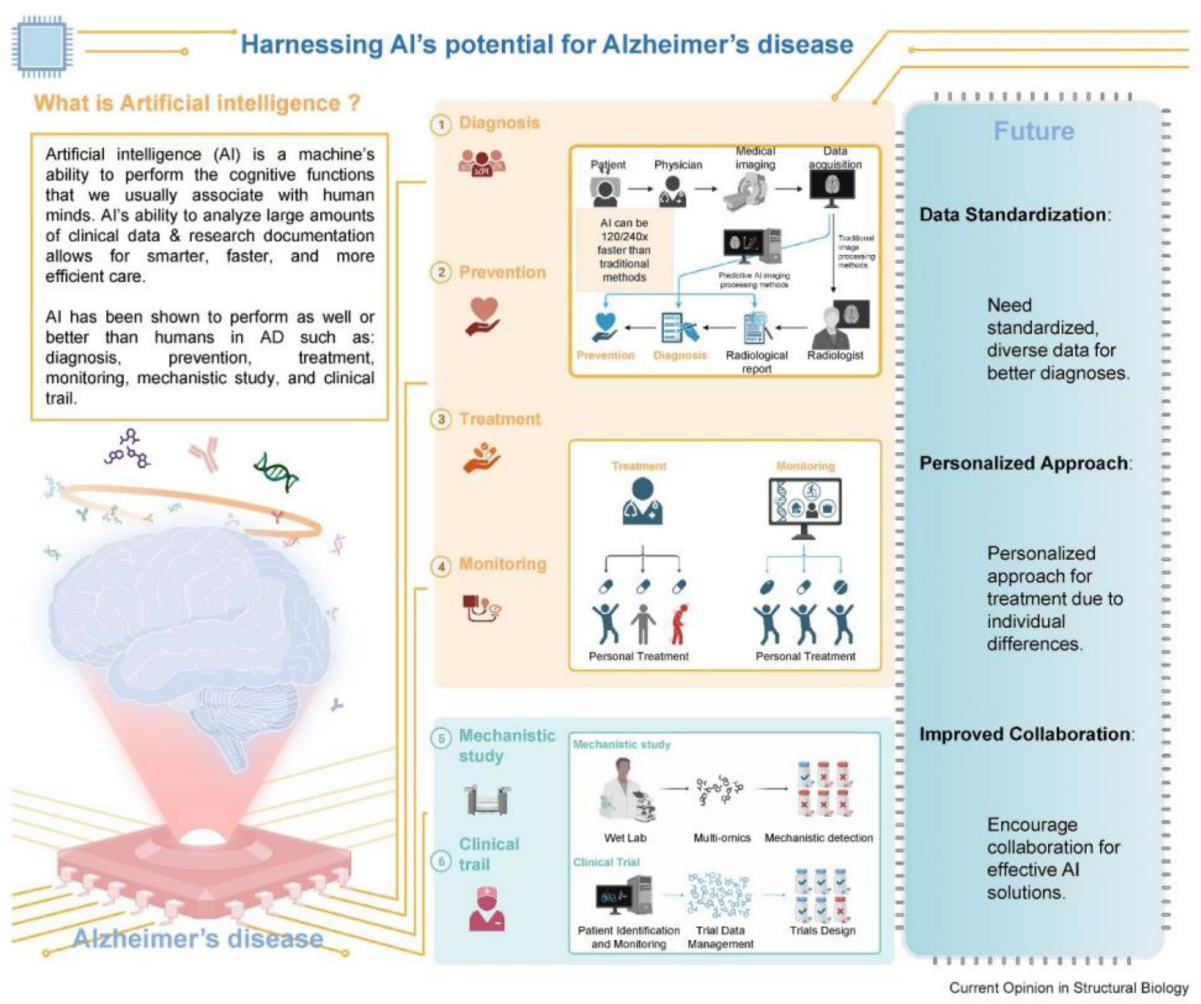
Conceptual summary of artificial intelligence applications in Alzheimer’s disease, including diagnosis, progression modeling, monitoring, therapy design, and clinical trial optimization. Source: Angelucci et al. (2024).

AI use in AD has several prospects to enhance diagnosis and therapy, as well as to expedite the identification of novel therapeutic targets; nonetheless, significant hurdles persist regarding its application (Figure 8).

### 7.2 Reinforcement learning

It is a branch of AI where an agent learns optimal strategies through trial and error by receiving feedback in the form of rewards. In the context of AD, RL has been employed to model disease progression and to develop adaptive intervention plans that evolve based on patient response over time. This makes RL particularly valuable compared to traditional supervised learning, which requires static labeled datasets and lacks the ability to adapt to longitudinal health data. By using RL, researchers can simulate complex, real-world scenarios and potentially enhance clinical decision-making in AD management.

## 8 Applications of AI in improving neurofunctionalities

### 8.1 Early detection and diagnosis

**FIGURE 9:**
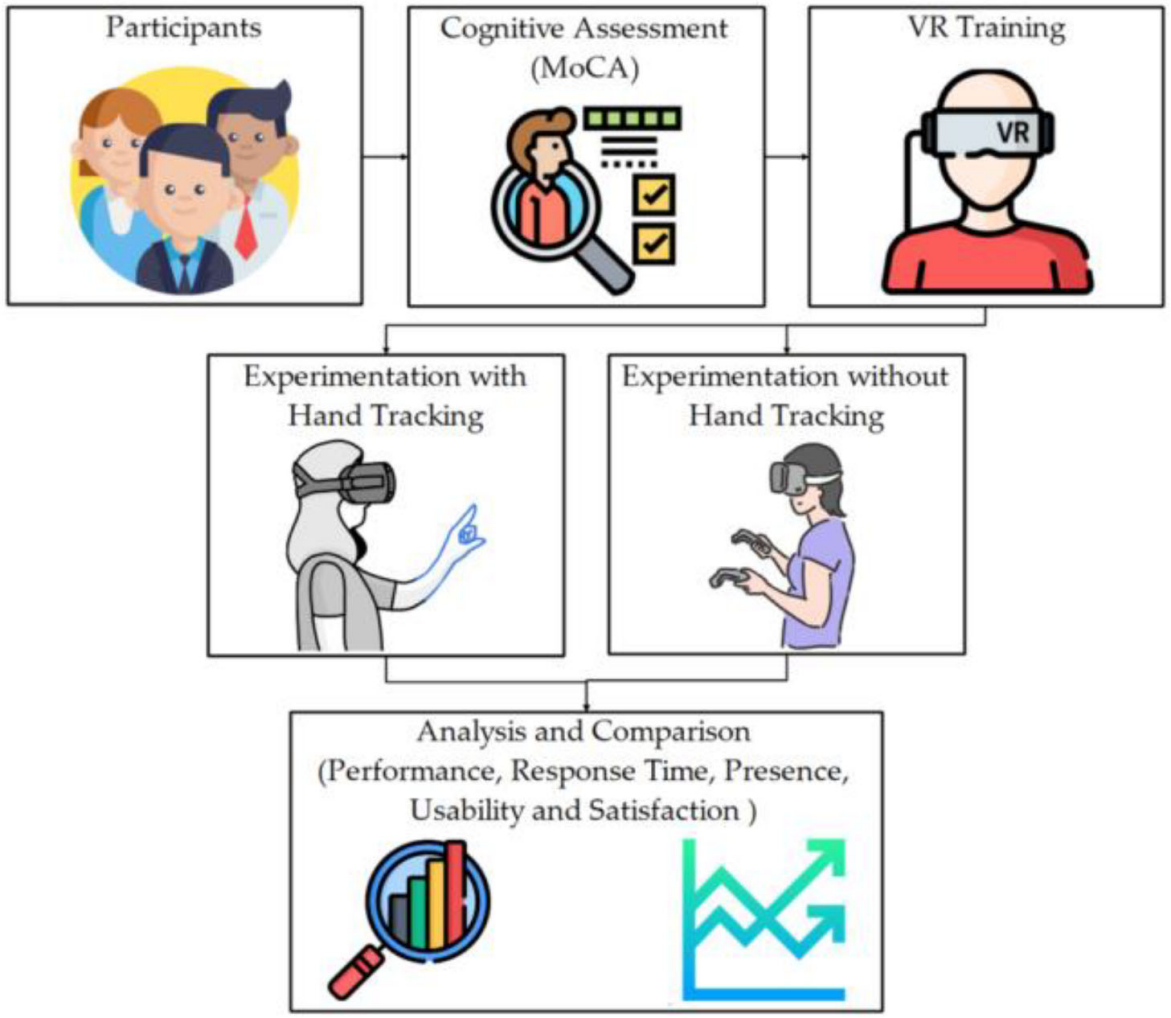
Schematic of a virtual reality (VR) and AI-based cognitive assessment workflow for Alzheimer’s patients. The system integrates cognitive task engagement, motion capture, and AI-based analytics to monitor neurofunctional changes. Adapted from Varela-Aldás et al. (2023).

It is evident that AI technologies in healthcare hold immense potential, especially in the early detection and diagnosis of chronic and progressive diseases like AD. One of the critical AI tools that is revolutionizing healthcare is Predictive modeling. It uses a well-trained AI algorithm that is capable of analyzing large datasets to identify individuals at risk of developing AD before clinical symptoms appear (Abadir et al., 2024). These models are possible upon incorporation and analysis of multiple layers and types of data, including genetic, imaging, and clinical information, to improve their performance and prediction accuracy. For instance, as reviewed recently by Angelucci et al. (2024) AI can analyze genetic markers and brain imaging data to identify early signs of AD, enabling timely interventions that may slow disease progression (Silva-Spínola et al., 2022) (Figure 9). They have further expanded on the significance of integrating the multimodal data as a critical aspect of early detection. Therefore, AI algorithms can provide a comprehensive view of a patient’s condition by combining information collected from different sources, such as MRI scans, PET images, blood tests, and cognitive assessments. This holistic approach not only enhances diagnostic accuracy but also helps to differentiate AD from other types of dementia (Subasi, 2020). Moreover, AI-driven diagnostic tools being non-invasive can further reduce the time and cost associated with traditional diagnostic methods, making early detection more accessible to the public as well (Zhang et al., 2023).

These practical implementations underscore the effectiveness of AI in real-world scenarios. For example, Jo et al. (2019) reported a diagnostic accuracy of 96.0% in AD classification and 84.2% in MCI progression prediction using deep learning models applied to MRI and PET data. Similarly, Chen et al. (2023) demonstrated the capability of natural language processing pipelines to extract cognitive and biomarker data from over 48,000 EHRs with an F1-score of 0.9059. Additional models such as D2NNLM (Orimaye et al., 2018) and multi-omics AI frameworks (Zhou et al., 2021) further validate the practical impact of AI in detecting early biomarkers and distinguishing AD subtypes in diverse populations.

Identifying reliable early biomarkers for AD is crucial for timely intervention and improved patient outcomes. Recent advances in AI, particularly in machine learning and deep learning, have made it possible to extract predictive biomarkers from complex, high-dimensional datasets such as neuroimaging, genomic, proteomic, and cognitive performance data. AI algorithms can detect subtle patterns that are imperceptible to human experts, thus enhancing early diagnostic accuracy.

A compelling example comes from Jo et al. (2019), who applied convolutional neural networks to neuroimaging data (MRI and PET) and achieved an accuracy of up to 96% in classifying AD, as well as 84.2% for predicting conversion from mild cognitive impairment to AD. This study highlighted how deep learning models could identify patterns in amyloid-beta and tau-related abnormalities even in the preclinical stages of AD.

Similarly, Chang et al. (2021) employed machine learning algorithms such as support vector machines (SVMs), in combination with blood and CSF biomarkers. Their findings demonstrated increased sensitivity and specificity when combining molecular biomarkers with AI, indicating that AI-enhanced platforms could outperform traditional diagnostic approaches that rely solely on clinical assessments.

In the study by Ning et al. (2018), MRI and genetic data were input into a neural network, which effectively classified subjects into cognitively normal, MCI, and AD groups. The model’s ability to predict future conversion from MCI to AD demonstrates AI’s utility in identifying early-stage biomarkers and patient risk profiling.

Zhou et al. (2021) further demonstrated the promise of AI in multi-omics biomarker discovery. Using a deep learning-based integration of transcriptomic and proteomic datasets, their model uncovered previously underexplored biomarkers like microRNAs (miRNAs) that correlate strongly with early-stage AD and cognitive decline trajectories.

Additionally, Chen et al. (2023) implemented a NLP-driven AI pipeline to mine EHRs, successfully extracting cognitive assessments and biomarker data (e.g., MMSE scores, CSF tau levels) from unstructured clinical notes. Their pipeline achieved an F1-score of 0.9059, showcasing the capability of AI to identify biomarkers from real-world data sources beyond controlled research settings.

These studies collectively underline the growing role of AI not just in data analysis but in early biomarker discovery for Alzheimer’s disease. By integrating diverse data modalities—such as structural MRI, PET imaging, genomic sequences, and cognitive assessments—AI platforms can build robust, predictive models that may eventually redefine diagnostic timelines and clinical trial eligibility.

### 8.2 Personalized treatment plans

With the advent of AI, the dream of personalized and precision medicine seems only a few more years away (Zhao et al., 2021). The boom of AI technologies is working tirelessly to revolutionize the development of personalized treatment plans for patient-specific AD. This has been made possible by analyzing individual patient data so that AI can analyze and recommend tailored pharmacological and non-pharmacological interventions. For example, we have recently witnessed the development of machine learning algorithms that can predict how patients will respond to specific medications based on their genetic profile, optimizing treatment efficacy and minimizing side effects (Mishra and Li, 2020). Personalized approaches like this ensure that patients receive the most appropriate care for their unique needs.

AI has further improved the patient monitoring aspect of healthcare as well, which used to be a significant limitation for consultants and physicians. AI has made this possible through advanced adaptive and responsive AI systems that are also being developed for continuous patient monitoring (da Rocha et al., 2024). These systems enable real-time tracking of patients’ cognitive and physical health that the concerned physician can utilize to adjust treatment plans as needed. The current revolution of intelligent wearable devices that are equipped with AI can efficiently monitor daily activities and ensure the detection of early signs of cognitive decline, thereby prompting timely interventions (Puri et al., 2022). Another feature of these AI-powered wearables is their cognitive training exercises while monitoring the progress of patients, aiming to enhance their cognitive function and overall quality of life.

### 8.3 Cognitive training and rehabilitation

Artificial Intelligence (AI) has emerged as a transformative tool in drug discovery and clinical trial design for Alzheimer’s disease (AD), addressing critical limitations of traditional methods such as prolonged timelines, high failure rates, and low translational success. Traditional AD drug development can span 12–15 years with a failure rate exceeding 99%, largely due to the complexity of AD pathophysiology and inadequate early-stage biomarkers (Cheng and Cummings, 2022). AI offers a data-driven alternative by integrating vast biological, chemical, and clinical datasets to accelerate hypothesis generation, target identification, and therapeutic optimization.

### 8.4 AI methodologies in drug discovery

#### 8.4.1 Deep Generative Models (DGMs)

Deep Generative Models, including VAEs and GANs, generate novel chemical structures with drug-like properties. For instance, Zeng et al. (2022) used deep generative modeling to screen chemical libraries and design small molecules targeting mitophagy, leading to the identification of two compounds that reversed memory deficits and reduced Aβ and tau pathology in AD mouse models.

#### 8.4.2 Network-based AI systems

Tools like AlzGPS (Alzheimer’s Gene-Phenotype-Signature Network System) integrate multi-omics data to identify disease modules and therapeutic targets through graph-based AI algorithms. This platform successfully predicted several drug repurposing candidates, such as pioglitazone and minocycline, which have since entered AD-related clinical evaluations (Kodam et al., 2023).

#### 8.4.3 Predictive ML models for ad target discovery

Supervised learning models (e.g., random forests, support vector machines) have been used to map gene expression patterns to disease phenotypes, identifying previously unrecognized targets like CD33 and TREM2, which are now under consideration for immunotherapeutic modulation (Fang et al., 2022).

#### 8.4.4 AI-driven clinical trial optimization

AI enhances trial efficiency by:

- Patient stratification based on genetic and phenotypic risk profiles.
- Predicting amyloid positivity using variables like family history, MRI features, and cognitive scores (Seo et al., 2022).
- Synthetic control arms generated via AI to reduce placebo group enrollment, thereby lowering costs and ethical concerns.

A notable example is the use of GANs by Islam and Zhang (2020), who created synthetic PET images for participants in AD, MCI, and control groups. These images were used to augment training datasets, resulting in a 10% increase in classification accuracy, thereby improving candidate identification for trial inclusion.

Furthermore, AI-guided eligibility screening has been shown to reduce screen failure rates by over 25%, particularly in trials requiring specific biomarker profiles (e.g., CSF Aβ42/tau ratios), which are costly and invasive to verify traditionally.

Table 2 summarizes various studies and computer-based cognitive improvement therapies for Alzheimer’s disease. The studies evaluated different Cognitive Tool like Interactive Computer-Based Cognitive Training, Structured Rehabilitative Software, and Virtual Reality Cognitive Training, showing significant improvements in task performance, neuropsychological domains, and cognitive functions, with some reporting sustained benefits and others noting limited general cognitive improvement.

**TABLE 2:** Studies and computer based cognitive improvement therapies.

Title of the study	Cognitive tool	Aim of the study	Main outcome and results	References
Interactive computer-training as a therapeutic tool in Alzheimer’s disease	Interactive Computer-Based Cognitive Training (ICT)	Evaluate the effectiveness of ICT in AD patients	AD patients showed significant improvement in task performance and reduction of mistakes over a 4 weeks training period. Training effects were sustained until follow-up 3 weeks later.	Hofmann et al., 2003
Computerized structured cognitive training in patients affected by early-stage Alzheimer’s disease is feasible and effective: a randomized controlled study	Structured Rehabilitative Software	Assess the neuropsychological effects of computerized cognitive training in early-stage AD patients	Significant improvement in various neuropsychological domains, with stable achievements after 6 months.	Cavallo et al., 2016
Interactive computer-based cognitive training in patients with Alzheimer’s disease	Interactive Computer-Based Program	Evaluate the use of an interactive computer-based program for AD patients	Patients needed less help, became faster, and made fewer mistakes after training. No evidence of general cognitive improvement.	Hofmann et al., 1996a
Virtual reality-based cognitive-motor training for middle-aged adults at high Alzheimer’s disease risk: a randomized controlled trial	Virtual Reality (VR) Cognitive Training	Examine the ability of VR cognitive training to improve cognition and cerebral blood flow in high AD risk individuals	Study focused on middle-aged individuals at high AD risk due to parental history.	Doniger et al., 2018
Computer-based cognitive training in Alzheimer’s disease patients	Computer-Based Program	Train AD patients using a computer-based program simulating everyday tasks	Improved training performance, high motivation, and positive acceptance of the training. No significant effects on general cognitive performance.	Hofmann et al., 1996b
Efficacy of cognitive training programs based on new software technologies in patients with Alzheimer-type dementia	Big Brain Academy (BBA)	Assess the efficacy of BBA compared to traditional cognitive training tools	BBA group showed significantly slower cognitive decline and greater decrease in depressive symptoms compared to other groups.	Fernández-Calvo et al., 2011
Evaluation of a computer-assisted errorless learning-based memory training program for patients with early Alzheimer’s disease in Hong Kong: a pilot study	Computerized Errorless Learning-Based Memory Training (CELP)	Compare CELP with therapist-led errorless learning and control group	Positive changes in cognitive function for CELP group, with remarkable changes in emotional/daily functions for therapist-led group.	Lee et al., 2013
Cognitive training and cognitive rehabilitation for mild to moderate Alzheimer’s disease and vascular dementia	Various Cognitive Training Programs	Evaluate the effectiveness of cognitive training and rehabilitation for AD and vascular dementia	Cognitive training showed no significant benefit. Cognitive rehabilitation showed promising results in participant and caregiver outcomes.	Bahar-Fuchs et al., 2019
Cognitive training interventions for patients with Alzheimer’s disease: a systematic review	Various Cognitive Training Programs	Review the effectiveness of cognitive training in AD patients	Positive effects reported in 24 out of 31 trials, mainly on global cognition and training-specific tasks. Little evidence of improved everyday functioning.	Kallio et al., 2017
Cognitive training for people with mild to moderate dementia	Various Cognitive Training Programs	Assess effects of cognitive training on cognitive and non-cognitive outcomes for dementia patients and caregivers	Moderate positive effects on global cognition and verbal semantic fluency. Benefits maintained in the medium term.	Kallio et al., 2017

### 8.5 Comparative analysis: AI-based vs. traditional diagnostic methods in AD

**Table:** 

Feature	Traditional diagnostic methods	AI-based diagnostic methods
Primary tools	Neuropsychological assessments, MRI/PET, CSF assays	Machine learning (ML), deep learning (DL), natural language processing (NLP), multimodal integration
Stage of detection	Typically moderate to late stages	Early/preclinical stages (e.g., eMCI, prodromal AD)
Biomarker detection	Manual interpretation of Aβ, tau, volumetric changes	Automated detection of structural, functional, and molecular patterns
Accuracy	∼70%–80% (clinician-dependent)	Up to 96% with DL (Jo et al., 2019)
Time and cost	High (invasive tests, multiple visits)	Lower (non-invasive imaging/speech analysis, EHR mining)
Scalability	Limited to specialized centres	High scalability using cloud-AI models and telehealth integration
Limitations	Delayed diagnosis, inter-rater variability	Black-box models, dependence on training data quality

While traditional diagnostic pathways have contributed significantly to our understanding of AD, they are limited in scalability, early detection, and interpretability. In contrast, AI systems—particularly those employing CNNs, transfer learning, and multimodal fusion—offer improved diagnostic performance, faster turnaround, and the ability to personalize assessments based on longitudinal data.

However, challenges remain for AI-driven diagnostics:

- Data bias due to imbalanced training datasets
- Regulatory and ethical concerns regarding explain ability and transparency
- Integration hurdles in clinical workflows (e.g., EHR interoperability)

Despite these challenges, ongoing advancements in explainable AI (XAI), federated learning for privacy-preserving modeling, and standardized validation protocols are steadily narrowing the gap between AI potential and routine clinical adoption.

### 8.6 Patient perceptions and experiences with AI-driven interventions

The integration of AI into Alzheimer’s disease care is not only a technological shift but also a human-centered transformation. Understanding how patients perceive and respond to AI-driven tools is essential for successful adoption.

Studies suggest that most patients and caregivers view AI positively, particularly when it enhances convenience, provides timely feedback, and supports independence. For example, AI-powered virtual assistants and cognitive training applications have been associated with increased engagement, reduced caregiver burden, and improved patient motivation (Ruggiano et al., 2021; Doniger et al., 2018). Patients often appreciate personalized interventions and adaptive learning systems that respond to their individual progress (McMurray et al., 2024).

However, some users express concerns regarding data privacy, loss of human interaction, and trust in automated decisions. These concerns highlight the importance of designing AI tools that are transparent, explainable, and used in complementary roles alongside healthcare professionals rather than as replacements.

Overall, patient experiences indicate that when AI tools are accessible, supportive, and ethically implemented, they can significantly enhance the quality of care and contribute positively to the patient journey in AD management.

## 9 Literature gaps and future research directions

Despite growing advancements in AI applications for Alzheimer’s disease, significant research and implementation gaps remain, particularly in underserved populations and low-resource regions.

### 9.1 Underrepresentation of diverse populations

Most AI models for AD diagnosis and management have been trained on datasets from high-income, Western countries, with limited inclusion of ethnically diverse or non-English-speaking populations. This lack of diversity raises concerns about algorithmic bias and limits the generalizability of these models to African, Latin American, South Asian, and rural elderly communities. There is a critical need to expand training datasets to include global and multicultural cohorts, particularly those with unique genetic, linguistic, or environmental risk factors for dementia.

### 9.2 Limited access to AI infrastructure

AI-driven diagnostics often require high-performance computing, reliable internet connectivity, and advanced imaging technologies (e.g., MRI, PET). These resources are largely unavailable in rural healthcare centers, low-income countries, and remote aging populations, resulting in an AI access divide. Research into lightweight, low-cost AI tools, including offline-capable mobile diagnostics, EEG-based algorithms, or speech-based cognitive assessments, is urgently needed to bridge this gap.

### 9.3 Insufficient real-world validation

While many AI models show high performance in experimental settings, relatively few have undergone prospective validation in clinical practice. More longitudinal studies, implementation trials, and post-deployment evaluations are needed to assess real-world impact, usability, and patient outcomes—especially in public health systems or primary care frameworks.

### 9.4 Lack of explainable AI and ethical transparency

Current models often function as “black boxes,” limiting clinician and patient trust. There is a need for research into explainable AI (XAI) approaches tailored for healthcare, ensuring that decisions are interpretable, clinically transparent, and ethically grounded.

### 9.5 Limited research on AI for preventive and non-pharmacological care

While AI in drug discovery is progressing, less attention has been given to its use in preventive interventions, behavioral health support, and low-tech cognitive rehabilitation, particularly for patients in early or prodromal AD stages. Expanding AI into lifestyle, nutrition, and caregiver support research may yield impactful, scalable strategies.

## 10 Conclusion

Alzheimer’s disease is a growing global health concern with significant impacts on patients, families, and healthcare systems. Traditional diagnostic and treatment methods face several challenges, highlighting the need for innovative solutions. AI offers promising advancements in early detection, personalized treatment, and cognitive rehabilitation for AD patients. Integrating AI tools within electronic health records can support real-time clinical decision-making, while AI-powered imaging platforms can streamline diagnosis and predictive analytics. However, several challenges must be addressed, including data interoperability, privacy concerns, lack of standard validation protocols, and clinician hesitation due to trust and usability issues. Solutions such as interoperable frameworks (e.g., FHIR), compliance with regulations like HIPAA/GDPR, development of explainable AI models, and cross-disciplinary training for medical professionals can support the seamless integration of AI. With collaborative efforts among government agencies, academic institutions, healthcare providers, and technology companies, AI can transform AD care through improved diagnosis, optimized therapy, and enhanced patient outcomes.
